# An outbreak of extensively drug-resistant and hypervirulent *Klebsiella pneumoniae* in an intensive care unit of a teaching hospital in Southwest China

**DOI:** 10.3389/fcimb.2022.979219

**Published:** 2022-09-13

**Authors:** Siyi Liu, Yinhuan Ding, Yifei Xu, Zhaoyinqian Li, Zhangrui Zeng, Jinbo Liu

**Affiliations:** Department of Laboratory Medicine, The Affiliated Hospital of Southwest Medical University, Luzhou, China

**Keywords:** *Klebsiella pneumoniae*, extensively drug-resistant, hypervirulent, tigecycline resistance, outbreak

## Abstract

Extensively drug-resistant and hypervirulent *Klebsiella pneumoniae* (XDR-hvKp) is a new problem for patients in Intensive Care Unit (ICU) and can become an even more severe threat if resistant to tigecycline, considered one of the ‘last lines of defense’ drugs. This study collected seven non-replicated tigecycline-resistant XDR-hvKp from seven patients and performed genome analysis and epidemiological investigation using whole genome equencing (WGS) and other methods. All strains in this study were identified as ST11-KL64 and showed high resistance to antibiotics such as β-lactams, aminoglycosides, quinolones, and tigecycline, and one strain was also resistant to colistin. All strains were determined to be hvKp by the results of serum resistance assay and *Galleria mellonella* infection models. All strains had resistance genes *bla*_CTX-M-65_,*bla*_KPC-2_,*bla*_LAP-2_,*bla*_TEM-1B_, *rmtB*, and *qnrS1* and virulence factors such as *rmpA*, *rmpA2*, and aerobactin (*iucABCD*, *iutA*). The expression of the AcrAB-TolC efflux pump was upregulated in all strains, and the expression levels of the gene *pmrK* was significantly upregulated in colistin-resistant strain DP compared to colistin-sensitive strain WT in this study. In conclusion, we described an outbreak caused by tigecycline-resistant XDR-hvKp in the ICU of a teaching hospital in southwest China. The spread of these superbugs poses a great threat to patients and therefore requires us to closely monitor these XDR-hvKp and develop relevant strategies to combat them.

## Introduction

*Klebsiella pneumoniae (K. pneumoniae)* is one of the most common opportunistic pathogens in hospitals, which causes pneumonia, sepsis, urinary tract infections, and other life-threatening diseases (Bengoechea and Sa Pessoa, 2019). At the same time, *K. pneumoniae* has attracted growing attention due to its rapid drug resistance and virulence evolution (Chang et al., 2021).

Classical *K. pneumoniae* (cKp) and hvKp are commonly divided into two pathogenic types for research based on their virulence, with hvKp being more virulent than cKp (Russo and Marr, 2019). HvKp can infect healthy individuals of any age, and it can cause hepatic abscesses in the absence of biliary tract infection or other symptoms; and can metastatically spread to multiple sites of the body, causing severe invasive infections were firstly reported in Taiwan and now prevalent mainly in the Asian Pacific Rim (Liu et al., 1986; Russo and Marr, 2019).The main virulence factors affecting hvKp are *rmpA*, *rmpA2*, aerobactin (*iucABCD, iutA*), salmochelin (*iroBCDNE*), *rmpC*, *rmpD*, and *peg344*. Briefly, *rmpA*, *rmpA2* regulate capsular polysaccharide (CPS) synthesis and mucoid phenotype; the siderophores aerobactin (*iucABCD*, *iutA*) and salmochelin (*iroBCDNE*) play an important role in infection; *rmpC*, *rmpD* and *peg344* are involved in virulence enhancement (Choby et al., 2020). Lately, some studies have found that mutations in the capsule-biosynthesis genes can also affect strains’ virulence (Ernst et al., 2020a; Morales-León et al., 2021).

HvKp can acquire antibiotic resistance by acquiring mobile genetic elements carrying antibiotic resistance genes or mutations in chromosomal genes under antibiotic pressure (Lee et al., 2017; Russo and Marr, 2019). At the same time multidrug-resistant (MDR) *K. pneumoniae* can enhance virulence by acquiring virulence or hybrid plasmids mediating multidrug-resistance and hypervirulence (Magiorakos et al., 2012; Lan et al., 2021). These conditions led to the emergence of carbapenem-resistant hypervirulent *K. pneumoniae* (CR-hvKp) which has both hypervirulent and multidrug-resistant phenotypes (Gu et al., 2018).

Colistin and tigecycline are among the few antibiotics treatments for carbapenem-resistant *K. pneumoniae* (CRKP) infections (Sheu et al., 2019). Unfortunately, pathogens can resist colistin and tigecycline through alterations of *mgrB*, mutations in the two-component regulatory systems (*pmrAB* and *phoPQ*), or disruption of regulatory genes encoding the efflux pumps (e.g., *ramR*, *ramA*, and *rarA*) (Cannatelli et al., 2014; Galani et al., 2021). If XDR-hvKp, poses a great threat to the patients in ICU (Xiong et al., 2021), were to become resistant to tigecycline, this would have even more severe consequences. We found that very little information is available on tigecycline-resistant CR-hvKp, and our search in PubMed resulted in only several papers about tigecycline-resistant CR-hvKp (Huang et al., 2018; Chen et al., 2021; Jin et al., 2021; Zhang et al., 2021). Hence, we need to keep an eye on these “superbugs”.

In this study, an outbreak of tigecycline-resistant XDR-hvKp in an ICU of a teaching hospital in southwest China, was investigated to elucidate the antibiotic resistance mechanisms and virulence factors of the strains, explore the evolution and transmission of XDR-hvKp in the clinical environment, and provide valuable information for monitoring and controlling these “superbugs”.

## Materials and methods

### Bacterial collection and analysis of epidemiological data

In February 2022, an outbreak of *K. pneumoniae* occurred in the ICU of a teaching hospital in southwest China. The antibiotic resistance mechanisms, virulence factors, and genetic correlates of strains isolated from seven non-replicated strains of *K. pneumoniae* obtained clinical specimens from seven patients from January 26, 2022, to February 24, 2022, were included in this study. The strains were identified by Matrix-assisted laser desorption/ionization-time of flight (MALDI-TOF) mass spectrometry (MS) (Bruker, Germany). The patient’s electronic medical record collected information on the patient’s gender, age, admission, diagnosis, antimicrobial therapy, and treatment outcome.

### Antimicrobial susceptibility testing

The Minimal inhibitory concentrations (MIC) of amikacin, gentamicin, cefepime, ceftazidime-avibactam, chloramphenicol, imipenem, meropenem, tetracycline, tigecycline, and colistin were determined using the microbroth dilution method. The susceptibility of the strains to other antibiotics was measured using the MicroScan Walkaway-96 system (Siemens, West Sacramento, CA, USA). *Pseudomonas aeruginosa* ATCC 27853 and *E. coli* ATCC 25922 were used as quality control strains. The strains’ susceptibility to tigecycline was interpreted according to the breakpoints set by the U.S. Food and Drug Administration (FDA) (≤2 mg/L for sensitive, 4 mg/L for intermediate, and ≥8 mg/L for resistant), and the remaining antibiotic results were interpreted according to the 2022 Clinical and Laboratory Standards Association (CLSI-M100-2022) guidelines (Pillar et al., 2008).

### String test

The strains were transferred to blood agar plates and incubated overnight. The colonies were touched with an inoculation loop and stretched outward, and the length of the sticky filaments pulled out was measured. A length larger than 5 mm was defined as a positive result, and the above operation was repeated three times for each strain (Yang et al., 2022).

### Serum resistance assay and *Galleria mellonella* infection modelstring test

The assay was performed according to the published experiment (Hughes et al., 1982; Podschun et al., 1993). Briefly, 25ul of a bacterial suspension at a concentration of 1.5×10^6^ CFU/mL was mixed with 75ul of healthy human serum and the mixture was incubated at 35°C. The baseline and the 1, 2 and 3 hour mixtures were diluted and inoculated on nutrient agar plates overnight. The number of colonies at each timepoint was counted, and the strains were graded (1, 2 for “highly sensitive”; 3, 4 for “moderately sensitive”; 5, 6 for “resistant”) as specified in the experimental method (Hughes et al., 1982; Podschun et al., 1993), depending on the results. Survival curves for serosensitivity were made according to the survival rate at each timepoint.

The experiments were performed according to a previously published method (Insua et al., 2013). Briefly, a suspension of 1×10^8^ CFU/mL was prepared from PBS-washed overnight cultures, and the suspension was diluted in a gradient to obtain a series of concentrations from 10^8^ to 10^5^. Ten microliters of suspension were injected into the *Galleria mellonella* larvae using a Hamilton syringe with a 30-gauge needle (15 biological replicates for each concentration). The larvaes were placed in the dark at 37°C with food and observed every 12 hours for three days and the number of surviving larvae was recorded. The experimental data were used to calculate the lethal dose 50 (LD_50_) according to the formula of Reed and Muench, and the results are expressed as log10 LD_50_ (Thakur and Fezio, 1981).

The hypervirulent *K. pneumoniae* NTUH-K2044 and the low-virulence ATCC 700603 were used as comparators. Both of the above experiments were repeated independently in triplicate.

### Pulsed-field gel electrophoresis (PFGE)

PFGE experiments were performed as described in the previous report (Han et al., 2013). Briefly, the strains in this study were inoculated on nutrient agar plates and incubated overnight at 37°C. We first digested bacterial suspensions (turbidity of 3.8-4.2) with proteinase K (TAKARA, Beijing) and then added agarose to obtain DNA gel blocks. Subsequently, slices of DNA gel blocks were digested with the restriction enzyme Xba I (TAKARA, Beijing). We performed electrophoresis at a voltage of 6 v/cm, with pulse parameters of 6–36s for 19 hours. We analyzed the results using BioNumerics (Version8.1, Applied maths, Inc.).

### Whole-genome sequencing (WGS) and bioinformatic analysis

Extraction and purification of total DNA from isolates incubated overnight in LB broth to logarithmic growth using the MagPure Bacterial DNA KF Kit (Magen). Quantification of total DNA was completed using the Qubit™ dsDNA HS Assay Kit (ThermoFisher) and Hieff NGS™ DNA Selection Beads (Shanghai). Library construction was completed using the NEB Next^®^ Ultra™ DNA Library Prep Kit for Illumina^®^ (NEB), followed by sequencing *via* the Illumina (NovaSeq 6000,USA) platform. Quality assessment of sequencing data was performed using FastQC (v 0.11.2), and validated data was obtained by trimming the sequencing data with Trimmomatic (v 0.36).

The Virulence Factor Database (VFDB) (http://www.mgc.ac.cn/VFs/) and BIGSdb Pasteur database (http://bigsdb.pasteur.fr) were used to obtain strains’ capsular serotypes and virulence genes. The IS finder (https://www-is.biotoul.fr/blast.php) was used to identify the type of insertion sequence (IS). The sequencing data were uploaded to the Center for Genomic Epidemiology (http://www.genomicepidemiology.org/) to obtain multilocus sequence typing (MLST), resistance genes, pore proteins, and plasmid replicon types for the strains. The genomes were compared using the Basic Local Alignment Search Tool (BLAST, https://blast.ncbi.nlm.nih.gov/Blast.cgi) and OAT software. A phylogenetic tree was constructed based on the Orthologous average nucleotide identity (OrthoANI) between the genomes.

Chromosomal mutations were identified by comparison with *K. pneumoniae* ATCC 13883 (accession number: JOOW01). *K. pneumoniae* genome (accession numbers LT174540 and JCMB01, respectively) was also used as a reference to identify mutations in the *rcsAB* and *lon* protease genes.

### Conjugation experiment

The strains were mixed as donors with the recipient bacteria (sodium azide-resistant *E. coli* J53) in LB broth in a 2:1 ratio and the mixture was incubated at 36°C for 24 hours. The transconjugants were screened on nutrient agar plates containing sodium azide (180 mg/L) and meropenem (4 mg/L) (Borgia et al., 2012). Transformants with *bla*_KPC_ (**Supplementary Table 1**) and resistance to meropenem were defined as transduction conjugates.

### Activity of efflux pump

In the presence of efflux pump inhibitor 1-(1-naphthylmethyl)-piperazine (NMP, 100mg/L), we determined the MIC of all strains to tigecycline. If the MIC value drops by a factor of 4 or more in the presence of epi, the efflux pump of the strain is significantly inhibited (Deng et al., 2014).

### Quantitative real-time PCR

Quantitative real-time PCR (RT-qPCR) was used to assess the expression levels of the *ramA*, *marA*, *soxS*, *acrA*, *acrB*, *acrR*, *phoP*, *phoQ*, *pmrD*, *pmrA*, *pmrB*, *pmrC*, and *pmrK* genes, using the primers in **Supplementary Table 1**. In brief, we extracted the RNA of strains in this study using the EasyPure RNA Kit (TransGen Biotech, Beijing, China). We used TransScript^®^ All-in-One First-Strand cDNA Synthesis SuperMix for qPCR (TransGen Biotech, Beijing, China) for reverse transcription. We performed the RT-qPCR assays using PerfectStart^®^ Green qPCR SuperMix (TransGen Biotech, Beijing, China). The 16S rRNA gene was used as an internal standard (Yang Y. et al., 2021), *K. pneumoniae* ATCC 13883 (expression = 1) was used as control (2^-ΔΔCT^ method). All experiments were performed for three replicates.

## Results

### Epidemiological investigation

**Figure 1:**
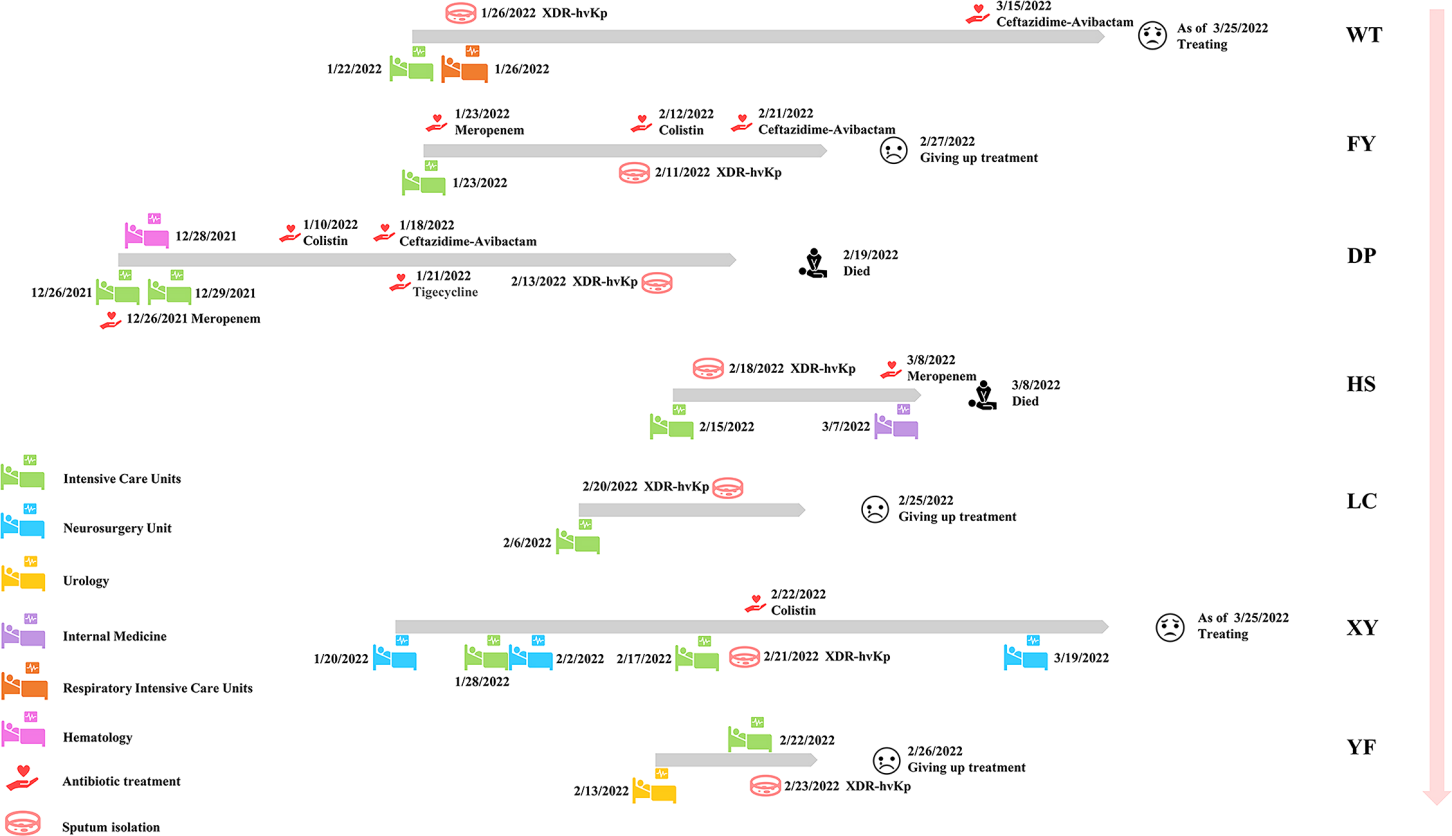
Epidemiology of seven patients in this study. Each grey broadband represents the timeline of one patient, the figure indicates the date of transfer of the patient to the new ward and the date of isolation of the strain as well as the date of the patient’s first use of this type of antibiotic in this investigation. The outcome of each patient is represented at the end of each grey broadband. The names of patients/strains are next to the pink broadband.

Seven non-replicated pathogens were isolated from the sputum of seven patients from December 26, 2021, to February 24, 2022, and identified as *K. pneumoniae* by MALDI-TOF MS. All patients had received invasive procedures (including tracheal intubation, catheter drainage, surgery, and puncture), and some had underlying diseases, such as tumors, hepatic impairment, and diabetes mellitus (**Table 1**). All patients had severe respiratory symptoms such as pulmonary infections. The first strain was isolated from the sputum specimen from patient WT on January 26, 2022. Patient WT was hospitalized in ICU on January 22, 2022, transferred to the Respiratory ICU on January 26, and was still under treatment in critical condition as of March 25. The last isolate was collected from the sputum of patient YF on February 23, 2022. A total of seven non-replicated strains from seven patients were obtained in this study, and all strains were isolated for the first time from each patient. As of March 25, 2022, among the seven patients, two had died, three abandoned treatment and were discharged from the hospital, and two were still undergoing treatment (**Figure 1**). Hospital workers took infection control measures promptly, so the outbreak was effectively controlled. Apart from these seven patients, no other *K. pneumoniae*-infected patients were found in the ICU and other departments for the time being. The follow-up epidemiological investigation is still in progress. The detailed molecular epidemiological investigation is described below.

**Table 1:** Clinical characteristics of patients with XDR-HvKp infection.

Patient	DP	FY	HS	LC	WT	XY	YF
**Variables**
Gender	Male	Male	Female	Male	Female	Male	Male
Age (years)	27	88	82	82	56	79	74
Department	ICU, hematology	ICU	ICU, internal medicine	ICU	ICU, respiratory ICU	ICU, neurosurgery unit	ICU, urology
Basic diseases	Acute myelocytic leukemia (M4), liver function damage	Liver function damage, cerebral infarction	Liver function damage, diabetes, kidney injury	Multiple organ failure	high blood pressure, anxiety disorders, depressive disorder	Diabetes, brain contusion	Renal pelvis tumor
Date of specimen collection: type	2/13/2022: Sputum	2/11/2022: Sputum	2/18/2022: Sputum	2/20/2022: Sputum	1/26/2022: Sputum	2/21/2022: Sputum	2/23/2022: Sputum
Infection type	Pneumonia, sepsis	Pneumonia, sepsis	Pneumonia, sepsis	Pneumonia, sepsis	Pneumonia	Pneumonia	Pneumonia
Therapeutic antimicrobial usage	MEM, VA, VRC, SXT, TGC, CZA, ATM, COL	MEM, CZA, ATM, VRC, TCP, CPS, COL	CPS, TZP, MEM	ONZ, CPS, MEM	CPS, VRC, LZD, CZA	TZP, VA, LVX, FLC, ETM, COL	CPS
Temperature (Tmax) (°C)	38.7	38.4	40	39	39.2	39.2	38.2
WBC (×10^9^/L)	1.15	2.28	15.6	6.6	10.24	5.30	20.62
PCT (ng/mL)	7.000	22.750	7.380	44.950	0.290	1.440	4.34
Invasive procedures^*^	Yes	Yes	Yes	Yes	Yes	Yes	Yes
Duration of ICU stay (days)	52	35	21	19	Unknown	Unknown	5
Outcomes	Died	Unknown	Died	Unknown	Treating	Treating	Unknown

ICU, Intensive Care Unit; TZP, Piperacillin-tazobactam; CZA, Ceftazidime-avibactam; ATM, Aztreonam; MEM, Meropenem; COL, Colistin; SXT, Trimethoprim-sulfamethoxazole; TGC, Tigecycline; VA, Vancomycin; LZD, Linezolid; CPS, Cefoperazone-sulbactam; LVX, Levofloxacin; ONZ, Ornidazole; FLC, Fluconazole; TCP, Teicoplanin; VRC, Voriconazole; ETM, Etimicin; Invasive procedures^*^, Including tracheal intubation, catheter drainage, surgery, and puncture.

### Antibiotic resistances

The results showed that all seven strains were resistant to β-lactams, quinolones, aminoglycosides, tetracyclines, sulfonamides, nitrofurantoin, and fosfomycin with almost identical MICs (**Table 2**). All strains were defined as XDR based on the drug sensitivity results. Notably, all six of the seven strains were sensitive to colistin, except for strain DP, which was resistant to colistin (MIC = 4 mg/L). All strains were sensitive to chloramphenicol and resistant to tigecycline with the same MIC (MIC = 8 mg/L), and no strain was found to be resistant to ceftazidime-avibactam.

**Table 2:** Antimicrobial susceptibility profiles of seven XDR-HvKp strains.

Isolates/patients	MIC (mg/L)																
	TZP	CZA	FEP	ATM	IPM	MEM	COL	GM	AN	TE	CIP	SXT	C	FOS	FM	TGC	TGC + NMP
WT	> 64/4	8/4	128	> 16	128	256	1	128	128	128	> 2	> 4/76	2	> 256	128	8	0.5
FY	> 64/4	8/4	128	> 16	128	256	1	128	128	128	> 2	> 4/76	2	> 256	128	8	0.5
DP	> 64/4	8/4	128	> 16	128	256	4	128	128	128	> 2	> 4/76	2	> 256	128	8	0.5
HS	> 64/4	8/4	128	> 16	128	256	1	128	128	128	> 2	> 4/76	2	> 256	128	8	0.5
XY	> 64/4	8/4	128	> 16	128	256	1	128	128	128	> 2	> 4/76	2	> 256	128	8	0.5
YF	> 64/4	8/4	128	> 16	128	256	1	128	128	128	> 2	> 4/76	2	> 256	128	8	0.5
LC	> 64/4	8/4	128	> 16	128	256	1	128	128	128	> 2	> 4/76	2	> 256	128	8	0.5

TZP, Piperacillin-tazobactam; CZA, Ceftazidime-avibactam; FEP, Cefepime; ATM, Aztreonam; IPM, Imipenem; MEM, Meropenem; COL, Colistin; GM, Gentamicin; AN, Amikacin; TE, Tetracycline; CIP, Ciprofloxacin; SXT, Trimethoprim-sulfamethoxazole; C, Chloramphenicol; FOS, Fosfomycin; FM, Nitrofurantoin; TGC, Tigecycline; NMP, 1-(1-Naphthylmethyl)-piperazine, 100 mg/L.

### Virulence phenotype

**Figure 2:**
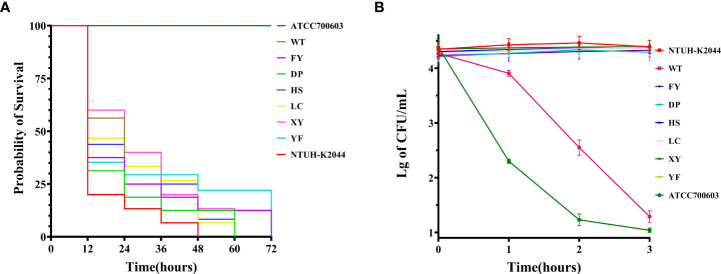
Virulence phenotypes of seven XDR-hvKp strains. **(A)** Survival curves of the *Galleria mellonella* larvae. **(B)** Activity of strains in serum from healthy human. The data are expressed as the Mean ± SE (standard error).In **(A, B)**, *K. pneumoniae* NTUH-K2044 and ATCC 700603 were used as positive control and negative control, respectively.

All strains could form a viscous filament larger than 5 mm. The serum resistance of strain WT was highly sensitive, whereas the other strains in this study were all resistant to serum (**Figure 2B**). The *Galleria mellonella* infection model showed an LD_50_ of 3.87 ± 0.17 (Mean ± SE) for *K. pneumoniae* NTHU-K2044 (**Supplementary Table 2**), which has no significant difference compared to the seven strains in this study (**Supplementary Figure 1**).

### Result of PFGE, analysis of whole-genome sequencing and construction of phylogenetic tree

**Figure 3:**
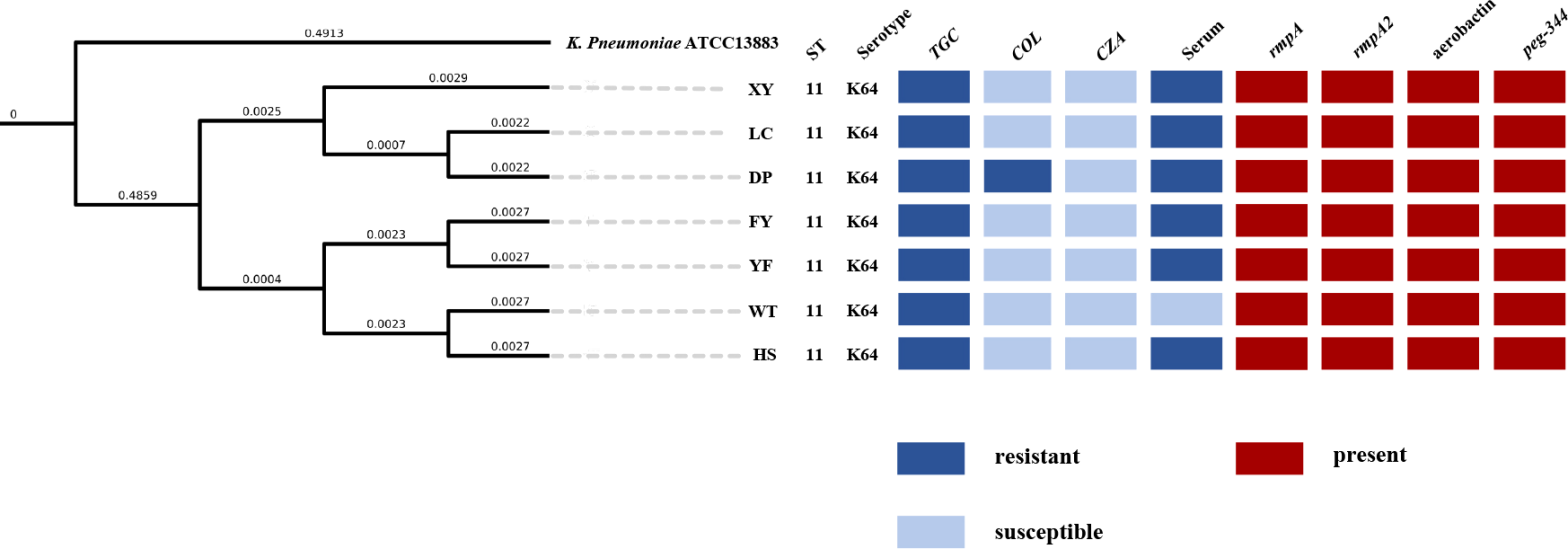
Phylogenetic tree of seven XDR-hvkp and *K. pneumoniae* ATCC 13883 based on OrthoANI values; *K. pneumoniae* ATCC 13883 was used as a control. The value on the branch, not the length of the branch in the graph, represents the real physical length of the branch.

**Figure 4:**
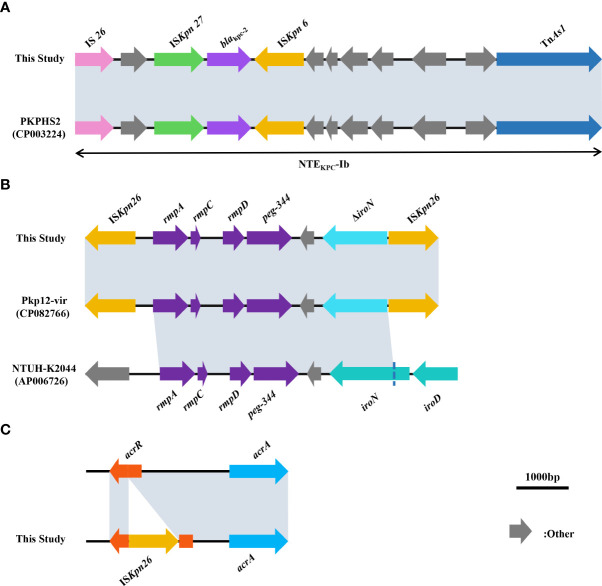
**(A)** Comparison of sequences surrounding *bla*_KPC-2_ between strains in this study and strain PKPHS2 (CP003224). **(B)** Comparison of sequences related to virulence among strains in this study, Pkp12-vir (CP082766), and NTUH-K2044 (AP006726). **(C)** Schematic representation of the insertion of the *acrR* gene by IS*kpn26*. In **(A–C)**, as all strains in this study showed almost identical results to those of the studies mentioned above, we show only a graphical representation of one of the seven strains at random. Grey shading indicates >99% identity between sequences.

The MLST of the seven non-replicated *K. pneumoniae* strains in this study was ST11, and the capsular serotype of all strains was KL64. The result of PFGE showed no difference in the bands of all strains (**Supplementary Figure 2**).The phylogenetic tree constructed by ANI showed that all strains were highly homologous (**Figure 3**). WGS analysis showed that all strains had *bla*_CTX-M-65_, *bla*_KPC-2_, *bla*_LAP-2_, *bla*_TEM-1B_ (β-lactam resistance genes), *rmtB* (aminoglycoside resistance gene), *fosA3* (fosfomycin resistance gene), *qnrS1* (quinolone resistance gene), *sul2* (sulphonamide resistance gene), *dfrA14* (trimethoprim resistance gene), *tet(A)* (tetracycline resistance gene), *ompk35*, *ompk37*, *ompk36* genes. Comparison with the sequence of *K. pneumoniae* ATCC 13883 (accession number: JOOW01) revealed that mutations in *gyrA*(Asp87Gly, Ser83Ile), *parC*(Ser80Ile) in the quinolone resistance determining region (QRDR) and *fosA* (Ile91Val) associated with fosfomycin resistance were present in all strains. We analyzed the gene sequences associated with tigecycline resistance including *tet(A)*, *ramR*, *ramA*, *acrR*, *acrA*, *acrB*, *acrD*, *marA*, *marR*, *rarA*, *robA*, *soxR*, *soxS*, *rpsj*, *tolc*, and *lon*. Comparison with *K. pneumoniae* ATCC 13883 revealed a mutation of stop194Lys in the *ramR* gene. And we also found that the *acrR* of all strains was truncated by the insertion of IS*Kpn26* (**Figure 4C**). Comparison with *Escherichia coli* plasmid RP1 tetracycline resistance determinants (GenBank accession number X00006) revealed the mutations of type 1 (Ile5Arg, Val55Met, Ile75Val, Thr84Ala, Ser201Ala, Phe202Ser, and Val203Phe), and Ala370Val in *tet(A)* in all strains. No other mutations were detected on other genes, and we did not find the genes like *tet(X)* or *tmexCD1-toprJ*-family in this study. We also analyzed the genes *pmrA*, *pmrB*, *pmrC*, *pmrD*, *phoP*, *phoQ*, *crrA*, *crrB*, *mgrB*, and *mcr*, associated with colistin resistance. The results showed that *pmrB* mutations such as Arg256Gly and Thr246Ala were present on all strains, but only one amino acid substitution was detected on *pmrB* (Thr157Pro) of the colistin-resistant strain DP, which differed from the other six strains (**Supplementary Table 3**). No other mutations were detected in the above genes, no insertions were detected on *mgrB*, and we also did not find the *mcr*-family genes.

All strains carried the virulence factors *rmpA*, *rmpA2*, *rmpC*, *rmpD*, aerobactin (*iucABCD*, *iutA*), entsiderophore, *iroE*, yersiniabactin. Besides, they also carried type III fimbriae, type I fimbriae, type IV pili and type VI secretion systems (T6SSs) related to colonization, adhesion, and microbial antagonism. No strains carried *iroBCD*, but all strains carried *iroN* truncated by IS*Kpn26*. Comparison with the genome of the *K. pneumoniae* (accession numbers LT174540 and JCMB01, respectively) revealed the presence of an amino acid substitution in *rcsA* (ser35Asn) in all strains, and no mutation was detected in the *lon* protease gene. Notably, the remaining six strains in this study had a shift mutation in the *wzc* region on the CPS gene cluster (Asn724Lys, Asn725Stop) compared to strain WT, which resulted in premature transcriptional termination.

For the gene environment of *bla*_KPC-2_, *bla*_KPC-2_ and the insertion sequence around *bla*_KPC-2_ together form a IS*Kpn27*- *bla*_KPC-2_- IS*Kpn6* structure, but no TN*4401* was found. Comparison with the NCBI data showed that *bla*_KPC-2_ and insertions upstream and downstream of *bla*_KPC-2_ in all strains formed the non-Tn*4401* element (NTE*_KPC_-Ib*) (**Figure 4A**). For the gene environment of *rmpA*, *rmpC* and *rmpD* and *peg-344* were surrounding *rmpA*, and there are two IS*Kpn26* at positions upstream of *rmpA* and downstream of *peg-344* respectively, *iroN* was truncated due to the insertion of IS*Kpn26* (**Figure 4B**). This region also had the same structure as the reported strain Pkp12-vir (GenBank accession number CP082766) by comparison with the NCBI data.

### Activity of efflux pump and results of RT-qPCR

**Figure 5:**
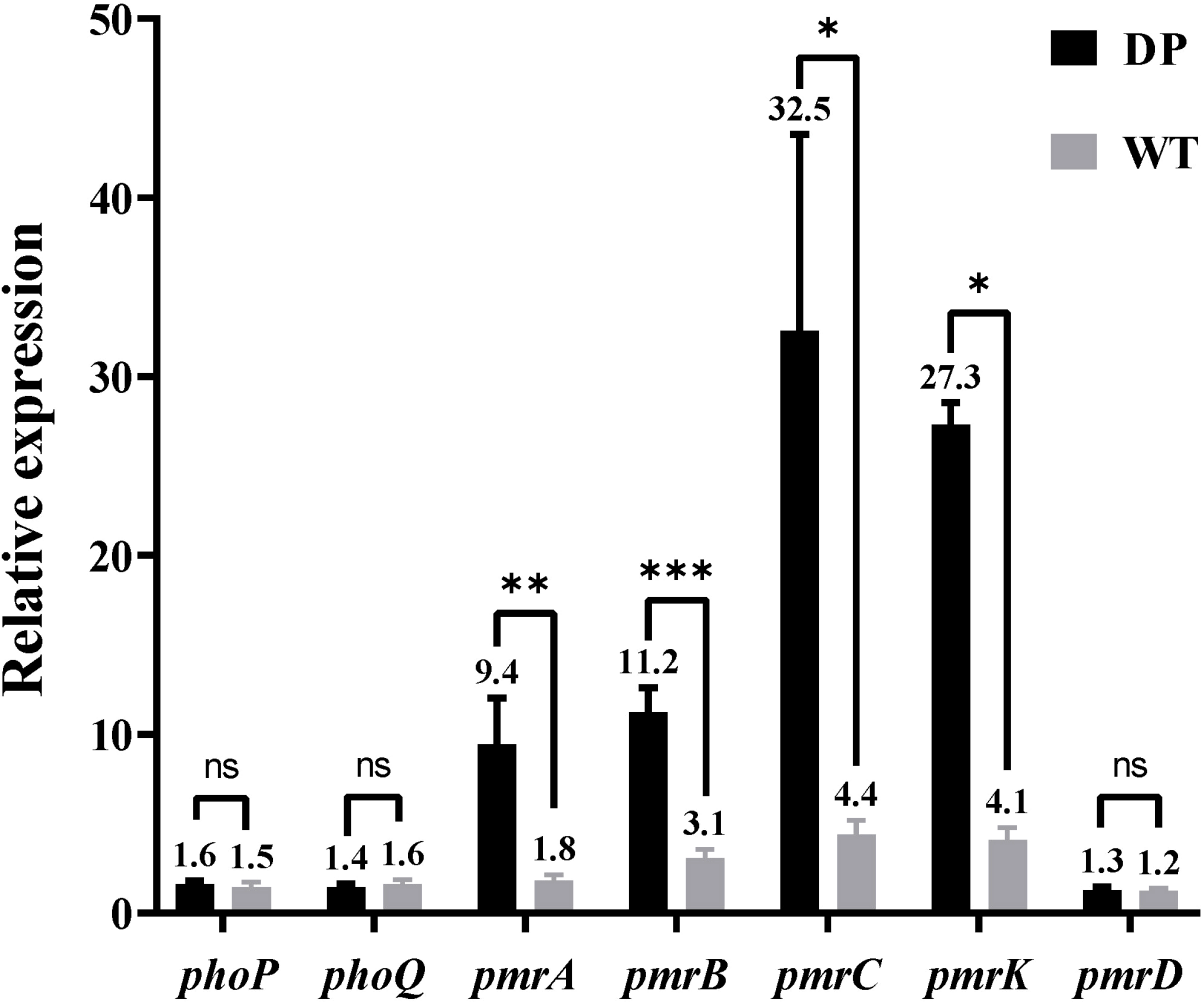
Comparison between the relative expression levels of the *phoP*, *phoQ*, *pmrC*, *pmrA*, *pmrB*, *pmrK*, and *pmrD* genes in strain DP and strain WT (Relative expression compared with *K. pneumoniae* ATCC13883, expression = 1). Values of relative expression are expressed as means and the standard deviation from experiments which were repeated in triplicate independently. *P<0.05; **P<0.01; ***P<0.001 by two-tailed unpaired *t* test; ns, no significance by two-tailed unpaired *t* test.

In the presence of NMP, the MIC of all strains for tigecycline decreased to 0.5 mg/L (**Table 1**). The results showed that NMP reversed the resistance of all strains to tigecycline in this study. Using *K. pneumoniae* ATCC 13883 as the control (expression = 1), RT-qPCR results showed that the expression of *acrA*, *acrB*, and *ramA* genes were significantly upregulated in all strains, *acrR* gene’s expression was significantly down-regulated, while the expression levels of *soxS* and *marA* were not statistically different compared to the control strain (**Table 3**). Expression levels of *pmrA*, *pmrB*, *pmrC*, and *pmrK* genes were significantly upregulated in colistin-resistant strain DP compared to colistin-sensitive strain WT, while the expression levels of *phoP*, *phoQ*, and *pmrD* did not show significant differences between strain WT and strain DP (**Figure 5**).

**Table 3:** Expression of *acrA*, *acrB*, *ramA*, *soxS*, *marA*, *acrR* of seven XDR-HvKp strains.

Isolation	Relative expression^a^					
	*acrA*	*acrB*	*ramA*	*soxS*	*marA*	*acrR*
ATCC13883	1	1	1	1	1	1
WT	2.90 ± 0.33^***^	2.78 ± 0.17^***^	9.40 ± 1.73^**^	1.11 ± 0.08^NO^	0.94 ± 0.07 ^NO^	0.20 ± 0.03^***^
FY	2.93 ± 0.30^***^	2.77 ± 0.31^***^	9.61 ± 1.22^***^	1.06 ± 0.15 ^NO^	1.04 ± 0.08 ^NO^	0.17 ± 0.03^***^
DP	2.74 ± 0.31^***^	2.32 ± 0.28^**^	7.33 ± 0.34^***^	1.07 ± 0.10 ^NO^	0.93 ± 0.15 ^NO^	0.19 ± 0.02^***^
HS	2.87 ± 0.55^**^	2.81 ± 0.35^***^	10.08 ± 0.77^***^	1.22 ± 0.39 ^NO^	0.90 ± 0.13 ^NO^	0.24 ± 0.01^***^
LC	2.47 ± 0.26^***^	2.71 ± 0.56^**^	10.54 ± 0.55^***^	1.13 ± 0.13 ^NO^	0.91 ± 0.14 ^NO^	0.14 ± 0.02^***^
XY	2.68 ± 0.33^***^	2.51 ± 0.23^***^	9.14 ± 1.20^***^	1.01 ± 0.16 ^NO^	1.11 ± 0.15 ^NO^	0.11 ± 0.02^***^
YF	2.62 ± 0.31^***^	2.90 ± 0.42^**^	11.23 ± 0.86^***^	1.02 ± 0.17 ^NO^	1.06 ± 0.16 ^NO^	0.31 ± 0.04^***^

a Relative expression compared with K. pneumoniae ATCC13883 (expression = 1); Results are means of 3 runs ± standard deviation.

^*^P<0.05; ^**^P<0.01; ^***^P<0.001 (p value vs. K. pneumoniae ATCC13883) by two-tailed unpaired t test; NO, no significance by two-tailed unpaired t test.

### Plasmid type and results of conjugation experiments

According to the results obtained from the Center for Genomic Epidemiology, all strains carried plasmids of the same plasmid type, IncFII, IncR, repB, ColRNAI, and IncHI1B (**Supplementary Table 3**). In this study, the plasmids of the tested strains were not transferred to *E. coli* J53 after several experiments.

## Discussion

Our study reported an outbreak of XDR-hvKp, which to our knowledge was the first report of an outbreak of XDR-hvKp in a hospital ICU in southwest China. The seven non-replicated strains isolated from the sputum of seven patients showed highly similar drug resistance (**Table 2**) and virulence phenotypes (**Figures 2A, B**), which, when combined with the results of PFGE (**Supplementary Figure 2**) and phylogenetic tree (**Figure 3**), suggested that the seven strains were closely related (Tenover et al., 1995; Jain et al., 2018). According to the chronological order of the patient’s ward and the patient’s infection, patient WT was in the ICU for only three days; therefore, the patient who may have cross-infected with patient WT during these three days was either patient FY or patient DP (**Figure 1**). Therefore, we speculated that strain WT might have been transmitted to patient FY or DP. Then strain FY or strain DP was transferred in an unknown manner to the remaining patients in this study. Another possibility is that strain FY or strain DP was transferred to patient WT and the rest of the patients in this study, and that patient WT left the ICU with strain WT to the respiratory ICU. However, the cause and the route of transmission of the outbreak in this study were still unknown and investigations were still ongoing.

The MLST of all strains in this study was ST11, the dominant clonal group of CRKP commonly found in China, and ST11 CRKP is also a high-risk clonal group with the ability to obtain hypervirulence-encoding plasmids (Liao et al., 2020). Recent studies had shown that ST11-KL64 was one of the most common types of ST11, the dominant clonal group of CR-hvKp in China, and was gradually replacing KL47. Furthermore, ST11-K64 CR-hvKp exhibited worrisome virulence and environmental survival rates (Liao et al., 2020; Zhou et al., 2020).

In terms of drug resistance of all strains, the patterns of antibiotic resistance in seven XDR strains were almost identical (**Supplementary Table 3**). The genes *bla*_CTX-M-65_, *bla*_KPC-2_, *bla*_LAP-2_, and *bla*_TEM-1B_ provide strains with β-lactams resistance (Galani et al., 2021). Among these genes *bla*_KPC-2_ was one of the most common carbapenemase genes in ST11 CRKP in China (Liao et al., 2020). The NTE*_KPC_-Ib* carried by all strains in this study (**Figure 4A**) was consistent with the NTE*_KPC_* possessed by strain PKPHS2 (CP003224) as previously reported (Chen et al., 2014), a mobile element that, like Tn*4401*, could play an important role in the transmission of *bla*_KPC_, more common in China and Brazil than Tn*4401*, the most prevalent in Europe and the United States (Yang X et al., 2021). None of the *bla*_KPC_-bearing plasmids from all strains in this study was successfully transferred into *E. coli* J53, which may be related to the host range and growth conditions of the strains, but did not exclude that the plasmids in this study could be transferred under other circumstances (Carattoli, 2009). In addition, mutations on *fosA* (Ile91Val) combined with the presence of *fosA3* in all strains could explain the resistance to Fosfomycin (Ito et al., 2017; Wang et al., 2022). The strains in this study were resistant to quinolones, caused by mutations in *gyrA* (Asp87Gly, Ser83Ile) and *parC* (Ser80Ile) of QRDR. All strains carried *qnrS1* which reduces susceptibility to quinolones (Fàbrega et al., 2009). All strains also carried *rmtB*, a common 16S rRNA methyltransferases (16S-RMTases) in Enterobacteriaceae, which could lead to a high-level of resistance to aminoglycosides, one of the therapeutic tools for CRKP (Doi et al., 2016). Unfortunately, tigecycline, one of the last resort treatments for XDR strains, was ineffective against all strains in this study (**Table 1**). Mutations in *tet(A)* (type 1 and Ala370Val) had all been shown to reduce the susceptibility of the strains to tigecycline (Chiu et al., 2017; Xu et al., 2021). Moreover, the *acrR* of the strains was also inserted by IS*Kpn26* (**Figure 4C**), which has been previously reported to activate the AcrAB-TolC efflux pump and reduce the strain’s susceptibility to tigecycline (Yang Y. et al., 2021). NMP can effectively inhibit the activity of the AcrAB-TolC efflux pump (Schuster et al., 2014). So, combining the results of RT-qPCR (**Table 3**) and efflux pump activity assays (**Table 2**), we can infer that the combination of *ramA* overexpression and the truncation of *acrR* by IS*Kpn26*, which resulted in the upregulation of AcrAB-TolC efflux pump, was the main cause of resistance to tigecycline in all strains in this study (Chiu et al., 2017). Incidentally, the mechanism of tigecycline resistance in all strains resulted from the accumulation of multiple mutations and was consistent, indirectly indicating a high degree of genetic relatedness between all strains. Notably, strain DP in this study was resistant to colistin, while the other strains were not; therefore, we speculate that the factor causing this phenomenon may be the development of resistance under the selective pressure of colistin (Huang et al., 2021). The strain DP showed amino acid substitutions in *pmrB* (Thr157Pro) that were not present in the remaining six strains. According to a previous study, Thr157Pro in *pmrB* could cause overexpression of *pmrCAB* and *pmrHFIJKLM* operons, resulting in colistin resistance (Jayol et al., 2014), which is consistent with the situation in this study (**Figure 5**). In addition, all strains had amino acid substitutions in *pmrB* (Arg256Gly and Thr246Ala) and although a previous study had shown that such mutations did not cause strains to be resistant to colistin, their effects might differ by genetic backgrounds, so more research is needed to demonstrate whether these mutations have other unknown promotive effects on the strains’ resistance to colistin (Cheng et al., 2015; Aires et al., 2016). Fortunately, all strains were susceptible to ceftazidime-avibactam (**Table 2**), and avibactam is a β-lactamase inhibitor that allows ceftazidime to retain its antibacterial activity in *Klebsiella pneumoniae* carbapenemase-producing Enterobacteriaceae by inhibiting Ambler class A β-lactamases so that ceftazidime-avibactam could be an option for treatment against the pathogens identified in this study (Zasowski et al., 2015).

In terms of virulence factors, all strains possess *rmpA*, *rmpA2*, *rmpC*, and *rmpD* capable of regulating the hypermucoviscosity (HMV) phenotype and CPS synthesis, which could explain the HMV of all strains (Wacharotayankun et al., 1993; Russo and Marr, 2019; Walker et al., 2020). The isolates in this study all possessed the siderophore genes aerobactin (*iucABCD, iutA*) and *iroE*, which help the strains to acquire iron in the human host (Choby et al., 2020), and *peg-344*, an inner membrane transporter whose role in virulence is not yet clear (Bulger et al., 2017). Besides, aerobactin plays a decisive role in the virulence of hvKp, and *peg-344*, *iucA*, *rmpA*, and *rmpA2* as markers to identify hvKp has been shown to have high accuracy (Russo et al., 2015; Russo et al., 2018). Therefore, based on the LD_50_, which was not statistically different from NTUH-K2044, and the virulence factors described above, it can be judged that all strains in this study were hvKp (Choby et al., 2020). In addition, the loss of *iroBCD* might be due to the insertion of IS*Kpn26*, which has been similarly reported previously (Kong et al., 2021). The amino acid substitution in *rcsA* (ser35Asn) of all strains was identical to the mutation reported in a Chilean’s study, such a condition could affect the synthesis of CPS, thereby enhancing the virulence of the strains (Morales-León et al., 2021). We also observed that the serum resistance of strain WT was different from other strains (**Figure 2B**). Previous studies had shown that mutations in the *wzc* gene affect the virulence of strains, so we hypothesized that mutations in the *wzc* of strain WT are responsible for the difference in serum resistance of strain WT from other strains, but further studies are needed to confirm this (Ernst et al., 2020b).

In conclusion, the seven tigecycline-resistant XDR-hvKp strains in this study showed worrying results regarding antibiotic resistance and virulence. We used WGS techniques to reveal the virulence and antibiotic resistance mechanisms of the strains in this study and to explore possible mechanisms of microevolution of the different strains and possible routes of transmission of the strains between patients in this outbreak. Our study provided a reliable basis for the developing infection control and prevention measures in the hospital and valuable information for clinical management. Our results also suggested the need for continued close surveillance of these superbugs and the need for healthcare workers to practice strict hand hygiene to prevent such events from occurring (Vermeil et al., 2019).

## Data availability statement

The datasets presented in this study can be found in online repositories. The names of the repository/repositories and accession number(s) can be found in the article/**Supplementary Material**.

## Author contributions

JL and ZZ conceived of and designed the study. SL, YX, and YD wrote this paper and contributed equally to this work. SL, YX, YD, and ZL performed the experiments. JL, ZZ, SL, and YX analyzed the data. All authors contributed to the article and approved the submitted version.

## Funding

This work was supported by the Science and Technology Project of Science & Technology Department of Sichuan Province under Grant [No. 2021YFS0329, No. 2022YFQ0093], Luxian and Southwest Medical University Cultivation Project [No. 2020LXXNYKD 04].

## Acknowledgments

We thank the curators of the Institute Pasteur MLST system (Paris, France) for the information about novel alleles, profiles, and/or isolates available at http://bigsdb.web.pasteur.fr. We thank the entire team of curators of GenBank databases for curating the data and making it publicly available at https://www.ncbi.nlm.nih.gov/.

## Conflict of interest

The authors declare that the research was conducted in the absence of any commercial or financial relationships that could be construed as a potential conflict of interest.

## Publisher’s note

All claims expressed in this article are solely those of the authors and do not necessarily represent those of their affiliated organizations, or those of the publisher, the editors and the reviewers. Any product that may be evaluated in this article, or claim that may be made by its manufacturer, is not guaranteed or endorsed by the publisher.
