# Supplementary material for: An outbreak of extensively drug-resistant and hypervirulent Klebsiella pneumoniae in an intensive care unit of a teaching hospital in Southwest China
Source: Front Cell Infect Microbiol. 2022 Sep 13;12:979219. doi: 10.3389/fcimb.2022.979219 (PMC9513609; doi:10.3389/fcimb.2022.979219)
Supplement: Supplementary file 1 [file DataSheet_1.docx]

Supplementary Material

# Supplementary Figures and Tables

**1.1 Supplementary Figures**





**Supplementary Figure 1.** Log10 LD_50_ (CFU) of NTUH-K2044 and seven XDR-hvKp strains in the *Galleria mellonella* infection model. ns, no significance (two-tailed unpaired *t* test).

**
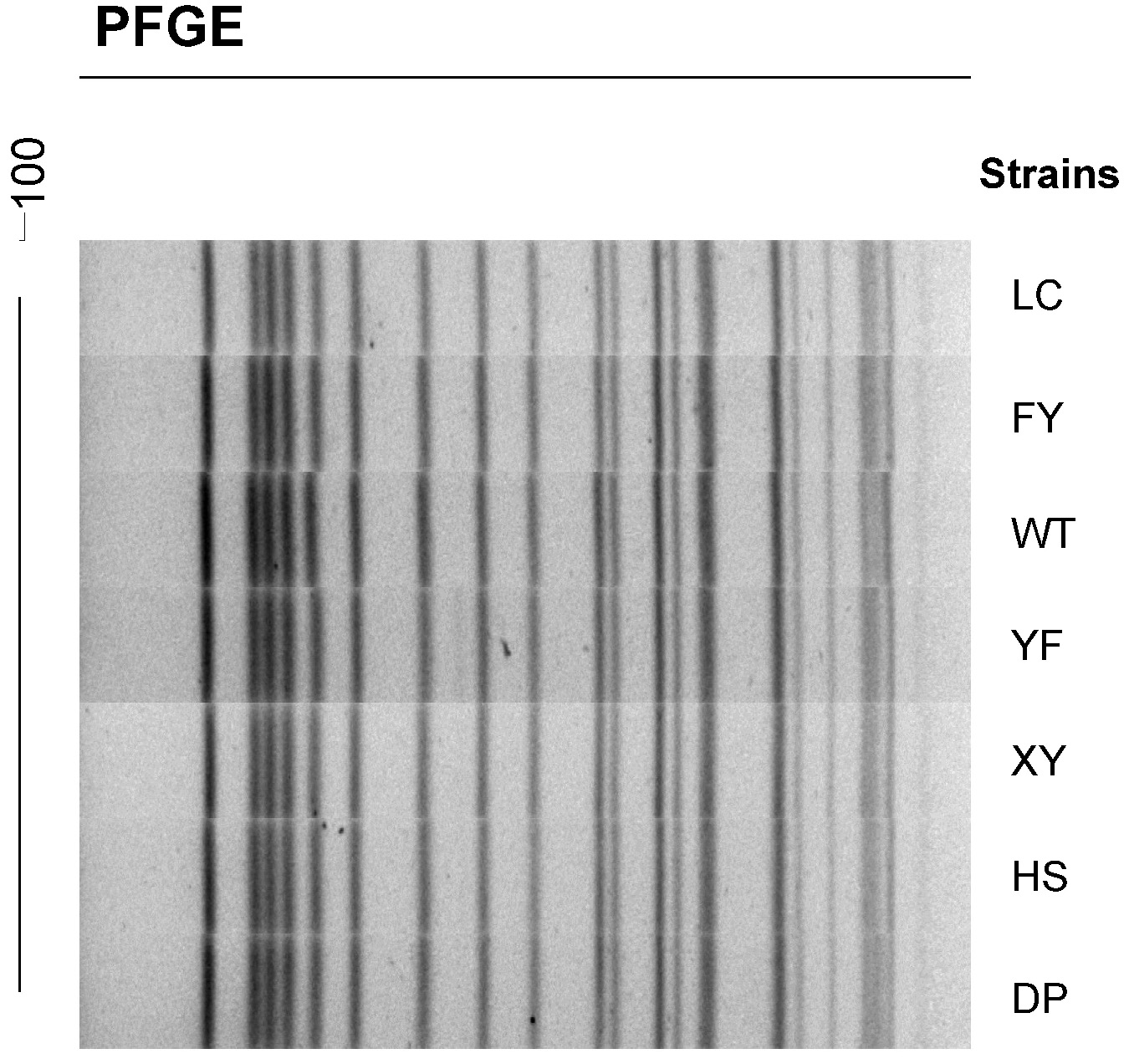
**

**Supplementary Figure 2.** PFGE results for seven XDR-hvKp strains in this study.

| Primer name | Sequence (5'-3') | Purpose | Gene | Reference or source |
| --- | --- | --- | --- | --- |
| *bla*_KPC_-F | ATGTCACTGTATCGCCGTCT | PCR | *bla*_KPC_ | ([Yan et al., 2021](#_ENREF_5)) |
| *bla*_KPC_-R | TTTTCAGAGCCTTACTGCCC | PCR |  |  |
| *ramA*-F | ATTTCCGCTCAGGTGATT | qRT-PCR | *ramA* | ([Sheng et al., 2014](#_ENREF_4)) |
| *ramA*-R | GTTGCAGATGCCATTTCG | qRT-PCR |  |  |
| *marA*-F | TAATGACGCCATCACTATCCA | qRT-PCR | *marA* | ([He et al., 2015](#_ENREF_2)) |
| *marA*-R | ATGTACTGGCCGAGGGAATG | qRT-PCR |  |  |
| *soxS*-F | TAGTCGCCAGAAAGTCAGGAT | qRT-PCR | *soxS* | ([He et al., 2015](#_ENREF_2)) |
| *soxS*-R | AGAAGGTTTGCTGCGAGACG | qRT-PCR |  |  |
| *acrR*-*F* | CTTGAAAGAGTGCATCGCGG | qRT-PCR | *acrR* | ([Yang et al., 2021](#_ENREF_6)) |
| *acrR*-*R* | GCATGCAGGTCGAACGAATC | qRT-PCR |  |  |
| *acrA*-F | GCCGGAAGTCGGTATTGTGA | qRT-PCR | *acrA* | ([Yang et al., 2021](#_ENREF_6)) |
| *acrA*-R | ACGCTTGACCGTCAGTTGAT | qRT-PCR |  |  |
| *acrB*-*F* | TGTCGGCGTCATTAACACCA | qRT-PCR | *acrB* | ([Yang et al., 2021](#_ENREF_6)) |
| *acrB*-*R* | TTTCAGGGCGCTGATCACAT | qRT-PCR |  |  |
| *phoP*-F | ATTGAAGAGGTTGCCGCCCGC | qRT-PCR | *phoP* | ([Cannatelli et al., 2013](#_ENREF_1)) |
| *phoP*-R | GCTTGATCGGCTGGTCATTCACC | qRT-PCR |  |  |
| *phoQ*-F | ATATGCTGGCGAGATGGGAAAACGG | qRT-PCR | *phoQ* | ([Cannatelli et al., 2013](#_ENREF_1)) |
| *phoQ*-R | CCAGCCAGGGAACATCACGCT | qRT-PCR |  |  |
| *pmrD*-F | GATCGCAGAGATTGAAGCCT | qRT-PCR | *pmrD* | ([Jayol et al., 2014](#_ENREF_3)) |
| *pmrD*-R | GCGTTGCGGATCTTCAAAGT | qRT-PCR |  |  |
| *pmrB*-F | TGCCAGCTGATAAGCGTCTT | qRT-PCR | *pmrB* | ([Jayol et al., 2014](#_ENREF_3)) |
| *pmrB*-R | TTCTGGTTGTTGTGCCCTTC | qRT-PCR |  |  |
| *pmrC*-F | GCGTGATGAATATCCTCACCA | qRT-PCR | *pmrC* | ([Jayol et al., 2014](#_ENREF_3)) |
| *pmrC*-R | CACGCCAAAGTTCCAGATGA | qRT-PCR |  |  |
| *pmrK*-F | AGTATCGGTCAGTGGCTGTT | qRT-PCR | *pmrK* | ([Jayol et al., 2014](#_ENREF_3)) |
| *pmrK*-R | CCGCTTATCACGAAAGATCC | qRT-PCR |  |  |
| *pmrA*-F | CAACCAGGGCGATAATGAAATCAGC | qRT-PCR | *pmrA* | This study |
| *pmrA*-R | CAGGGCATACTCTTTCGGCGTAAG | qRT-PCR |  |  |
| 16S rRNA-F | CGGTGAATACGTTCYCGG | qRT-PCR | 16S rRNA | ([Yang et al., 2021](#_ENREF_6)) |
| 16S rRNA-R | GGWTACCTTGTTACGACTT | qRT-PCR |  |  |

**1.2 Supplementary Tables.**

**Supplementary Table 1.** Primers used in this study.

Cannatelli, A., D'andrea, M.M., Giani, T., Di Pilato, V., Arena, F., Ambretti, S., Gaibani, P., and Rossolini, G.M. (2013). In vivo emergence of colistin resistance in Klebsiella pneumoniae producing KPC-type carbapenemases mediated by insertional inactivation of the PhoQ/PhoP mgrB regulator. *Antimicrob Agents Chemother* 57**,** 5521-5526.

He, F., Fu, Y., Chen, Q., Ruan, Z., Hua, X., Zhou, H., and Yu, Y. (2015). Tigecycline susceptibility and the role of efflux pumps in tigecycline resistance in KPC-producing Klebsiella pneumoniae. *PLoS One* 10**,** e0119064.

Jayol, A., Poirel, L., Brink, A., Villegas, M.V., Yilmaz, M., and Nordmann, P. (2014). Resistance to colistin associated with a single amino acid change in protein PmrB among Klebsiella pneumoniae isolates of worldwide origin. *Antimicrob Agents Chemother* 58**,** 4762-4766.

Sheng, Z.K., Hu, F., Wang, W., Guo, Q., Chen, Z., Xu, X., Zhu, D., and Wang, M. (2014). Mechanisms of tigecycline resistance among Klebsiella pneumoniae clinical isolates. *Antimicrob Agents Chemother* 58**,** 6982-6985.

Yan, R., Lu, Y., Zhu, Y., Lan, P., Jiang, S., Lu, J., Shen, P., Yu, Y., Zhou, J., and Jiang, Y. (2021). A Sequence Type 23 Hypervirulent Klebsiella pneumoniae Strain Presenting Carbapenem Resistance by Acquiring an IncP1 bla (KPC-2) Plasmid. *Front Cell Infect Microbiol* 11**,** 641830.

Yang, Y., Yang, Y., Chen, G., Lin, M., Chen, Y., He, R., Galvão, K.N., El-Gawad El-Sayed Ahmed, M.A., Roberts, A.P., Wu, Y., Zhong, L.L., Liang, X., Qin, M., Ding, X., Deng, W., Huang, S., Li, H.Y., Dai, M., Chen, D.Q., Zhang, L., Liao, K., Xia, Y., and Tian, G.B. (2021). Molecular characterization of carbapenem-resistant and virulent plasmids in Klebsiella pneumoniae from patients with bloodstream infections in China. *Emerg Microbes Infect* 10**,** 700-709.

**Supplementary Table 2.** Results of serum resistance assay and Galleria mellonella infection model.

| **Isolation** | **Serum resistance (** **log10-transformed values, Mean ± SE)** | | | | **Grade^*^** | **Log10 LD_50_(CFU)** | |
| --- | --- | --- | --- | --- | --- | --- | --- |
|  | **0h** | **1h** | **2h** | **3h** |  | **Mean ± SD** | ***p value vs*. NTUH-K2044** |
| WT | 4.27 ± 0.08 | 3.91 ± 0.06 | 2.55 ± 0.14 | 1.29 ± 0.11 | 2 | 3.90 ± 0.11 | NO (0.53) |
| FY | 4.23 ± 0.16 | 4.26 ± 0.14 | 4.30 ± 0.13 | 4.33 ± 0.12 | 6 | 3.99 ± 0.11 | NO (0.56) |
| DP | 4.21 ± 0.09 | 4.28 ± 0.08 | 4.33 ± 0.09 | 4.29 ± 0.16 | 5 | 4.16 ± 0.16 | NO (0.09) |
| HS | 4.30 ± 0.09 | 4.34 ± 0.06 | 4.36 ± 0.05 | 4.38 ± 0.04 | 6 | 4.02 ± 0.07 | NO (0.23) |
| LC | 4.28 ± 0.14 | 4.32 ± 0.13 | 4.36 ± 0.14 | 4.39 ± 0.13 | 6 | 4.07 ± 0.13 | NO (0.17) |
| XY | 4.35 ± 0.10 | 4.37 ± 0.10 | 4.39 ± 0.09 | 4.39 ± 0.12 | 6 | 4.09 ± 0.09 | NO (0.06) |
| YF | 4.30 ± 0.02 | 4.33 ± 0.06 | 4.38 ± 0.05 | 4.40 ± 0.05 | 6 | 4.00 ± 0.06 | NO (0.25) |
| NTUH-K2044 | 4.35 ± 0.13 | 4.43 ± 0.11 | 4.46 ± 0.12 | 4.39 ± 0.11 | 5 | 3.87 ± 0.17 | NA |
| ATCC700603 | 4.38 ± 0.16 | 2.30 ± 0.04 | 1.23 ± 0.11 | 1.04 ± 0.04 | 1 | NA | NA |

SE, standard error; SD, standard deviation; NA, not applicable; CFU=colony forming unit; NO, no significance by two-tailed unpaired *t* test. Grade*:1, 2 for “highly sensitive”; 5, 6 for “resistant”.

**Supplementary Table 3.** Genomic characterization of seven XDR-HvKp in this study.

| Strains | Beta-lactam | Tetracycline | Fluoroquinolone | Aminoglycoside | Others | Hypervirluence factors | *pmrB* | *ramR* | Predicted plasmid elements |
| --- | --- | --- | --- | --- | --- | --- | --- | --- | --- |
| WT | *bla*_CTX-M-65_, *bla*_KPC-2_, *bla*_LAP-2_, *bla*_TEM-1B_ | *tet(A)* (Type 1, A370V) | *qnrS1*, *gyrA* (D87G,S83I), *parC* (S80I) | *rmtB* | *fosA* (I91V), *fosA3*, *dfrA14*, *sul2* | *rmpA, rmpA2, rmpC, rmpD, iucABCD, iutA, fyuA, irp1, irp2, ybtAEPQSTUX, iroE* | T157P R256G T246A | stop194K | IncFII, IncR, repB, ColRNAI, IncHI1B |
| FY | *bla*_CTX-M-65_, *bla*_KPC-2_, *bla*_LAP-2_, *bla*_TEM-1B_ | *tet(A)* (Type 1, A370V) | *qnrS1*, *gyrA* (D87G,S83I), *parC* (S80I) | *rmtB* | *fosA* (I91V),  *fosA3*, *dfrA14*, *sul2* | *rmpA, rmpA2, rmpC, rmpD, iucABCD, iutA, fyuA, irp1, irp2, ybtAEPQSTUX, iroE* | R256G  T246A | stop194K | IncFII, IncR, repB, ColRNAI, IncHI1B |
| DP | *bla*_CTX-M-65_, *bla*_KPC-2_, *bla*_LAP-2_, *bla*_TEM-1B_ | *tet(A)* (Type 1, A370V) | *qnrS1*, *gyrA* (D87G,S83I), *parC* (S80I) | *rmtB* | *fosA* (I91V),  *fosA3*, *dfrA14*, *sul2* | *rmpA, rmpA2, rmpC, rmpD, iucABCD, iutA, fyuA, irp1, irp2, ybtAEPQSTUX, iroE* | R256G  T246A | stop194K | IncFII, IncR, repB, ColRNAI, IncHI1B |
| HS | *bla*_CTX-M-65_, *bla*_KPC-2_, *bla*_LAP-2_, *bla*_TEM-1B_ | *tet(A)* (Type 1, A370V) | *qnrS1*, *gyrA* (D87G,S83I), *parC* (S80I) | *rmtB* | *fosA* (I91V),  *fosA3*, *dfrA14*, *sul2* | *rmpA, rmpA2, rmpC, rmpD, iucABCD, iutA, fyuA, irp1, irp2, ybtAEPQSTUX, iroE* | R256G  T246A | stop194K | IncFII, IncR, repB, ColRNAI, IncHI1B |
| XY | *bla*_CTX-M-65_, *bla*_KPC-2_, *bla*_LAP-2_, *bla*_TEM-1B_ | *tet(A)* (Type 1, A370V) | *qnrS1*, *gyrA* (D87G,S83I), *parC* (S80I) | *rmtB* | *fosA* (I91V),  *fosA3*, *dfrA14*, *sul2* | *rmpA, rmpA2, rmpC, rmpD, iucABCD, iutA, fyuA, irp1, irp2, ybtAEPQSTUX, iroE* | R256G  T246A | stop194K | IncFII, IncR, repB, ColRNAI, IncHI1B |
| YF | *bla*_CTX-M-65_, *bla*_KPC-2_, *bla*_LAP-2_, *bla*_TEM-1B_ | *tet(A)* (Type 1, A370V) | *qnrS1*, *gyrA* (D87G,S83I), *parC* (S80I) | *rmtB* | *fosA* (I91V),  *fosA3*, *dfrA14*, *sul2* | *rmpA, rmpA2, rmpC, rmpD, iucABCD, iutA, fyuA, irp1, irp2, ybtAEPQSTUX, iroE* | R256G  T246A | stop194K | IncFII, IncR, repB, ColRNAI, IncHI1B |
| LC | *bla*_CTX-M-65_, *bla*_KPC-2_, *bla*_LAP-2_, *bla*_TEM-1B_ | *tet(A)* (Type 1, A370V) | *qnrS1*, *gyrA* (D87G,S83I), *parC* (S80I) | *rmtB* | *fosA* (I91V),  *fosA3*, *dfrA14*, *sul2* | *rmpA, rmpA2, rmpC, rmpD, iucABCD, iutA, fyuA, irp1, irp2, ybtAEPQSTUX, iroE* | R256G  T246A | stop194K | IncFII, IncR, repB, ColRNAI, IncHI1B |

G: Gly; D: Asp; S: Ser; I: Ile; V: Val; R: Arg; T: Thr; A: Ala; Q: Gln; P: Pro; M: Met; F: Phe; K: Lys; Type 1: Ile5Arg, Val55Met, Ile75Val, Thr84Ala, Ser201Ala, Phe202Ser, Val203Phe.
